# Development of Essential Oil-Loaded Polymeric Nanocapsules as Skin Delivery Systems: Biophysical Parameters and Dermatokinetics Ex Vivo Evaluation

**DOI:** 10.3390/molecules28207142

**Published:** 2023-10-18

**Authors:** Perla Giovanna Silva-Flores, Sergio Arturo Galindo-Rodríguez, Luis Alejandro Pérez-López, Rocío Álvarez-Román

**Affiliations:** 1 Departamento de Embriología, Facultad de Medicina, Universidad Autónoma de Nuevo León, Monterrey 64460, Mexico; psilva.me0208@uanl.edu.mx; 2 Departamento de Química Analítica, Facultad de Ciencias Biológicas, Universidad Autónoma de Nuevo León, San Nicolás de los Garza 66455, Mexico; sergio.galindord@uanl.edu.mx; 3 Departamento de Química Analítica, Facultad de Medicina, Universidad Autónoma de Nuevo León, Monterrey 64460, Mexico; luis.perezlp@uanl.edu.mx

**Keywords:** nanocapsules, skin delivery, *Rosmarinus officinalis*, *Lavandula dentata*, tape stripping

## Abstract

Essential oils (EOs) are natural antioxidant alternatives that reduce skin damage. However, EOs are highly volatile; therefore, their nanoencapsulation represents a feasible alternative to increase their stability and favor their residence time on the skin to guarantee their effect. In this study, EOs of *Rosmarinus officinalis* and *Lavandula dentata* were nanoencapsulated and evaluated as skin delivery systems with potential antioxidant activity. The EOs were characterized and incorporated into polymeric nanocapsules (NC-EOs) using nanoprecipitation. The antioxidant activity was evaluated using the ferric thiocyanate method. The ex vivo effects on pig skin were evaluated based on biophysical parameters using bioengineering techniques. An ex vivo dermatokinetic evaluation on pig skin was performed using modified Franz cells and the tape-stripping technique. The results showed that the EOs had good antioxidant activity (>65%), which was maintained after nanoencapsulation and purification. The nanoencapsulation of the EOs favored its deposition in the stratum corneum compared to free EOs; the highest deposition rate was obtained for 1,8-cineole, a major component of *L. dentata*, at 1 h contact time, compared to *R. officinalis* with a major deposition of the camphor component. In conclusion, NC-EOs can be used as an alternative antioxidant for skin care.

## 1. Introduction

Essential oils (EOs) are natural products obtained from aromatic plants using vapor distillation or hydrodistillation; they are made up of hydrophobic and volatile molecules [1,2] and may contain over 20–100 components within an interval of different concentrations, of which terpenes and phenylpropanoids are the most predominant [3]. Owing to their complex chemical compositions, EOs are widely used in the cosmetic, pharmaceutical, therapeutic, and food industries [1,4]. The EOs of *Rosmarinus officinalis* and *Lavandula dentata* are obtained from aromatic plants belonging to the *Lamiaceae* family and have been widely used in traditional medicine owing to their antioxidant, antibacterial, and antifungal properties [5,6,7]. Thus, the antioxidant properties of EOs play a fundamental role in oxidative stress because of the inherent capacity of some of their main components to stop or delay the aerobic oxidation of organic matter (*for example, membrane lipids*) [8].

The skin is the largest organ of the human body and plays a protective role; therefore, it is constantly exposed to endogenous oxidative stress (e.g., peroxidases, cyclooxygenases, and lipid oxygenase) and exogenous (e.g., ultraviolet radiation, chemicals, and pollution) sources [9]. Oxidative stress is caused by the overproduction of free radicals and reactive oxygen species (ROS) in biological systems, which cannot be completely eliminated by the antioxidant system and may cause issues, such as premature aging or skin problems [10]. The skin is an excellent natural barrier due to a “brick–mortar” structure, where the “bricks” are non-nucleated stratum corneum (SC) cells and the “mortar” is a lipid-rich extracellular matrix [10,11]. The skin protects the body from excessive water loss and pathogen penetration. Therefore, maintaining skin homeostasis to prevent and/or minimize damage caused by free radicals is necessary, for which the use of external antioxidant agents has been proposed [3,12]. The search of natural antioxidants that allow to substitute synthetic antioxidants has led to several studies about the antioxidant potential of EOs [8]. However, EOs are unstable owing to their chemical complexity and high volatility. Therefore, exploring methods for enhancing their stability and efficacy in skin care applications is required [1,13]. In this regard, nanoencapsulation has emerged as a relevant alternative that can improve the stability, protect EOs against degradation, and control their release [14].

Nanoparticles are colloidal systems with a particle size of <1 mm and can be classified as nanocapsules (NCs) or nanospheres according to their composition. They enable the encapsulation of lipophilic compounds, such as EOs [15]. In particular, polymeric nanocapsules are of special interest with regard to skin applications because of their nanometric size, which makes it possible to increase the time of residence and direct contact with the skin, which favors the release of EOs on the SC and appendices of the skin. This could increase the quantity of the active compound that reaches the targeted action site and therefore its biological activity [16].

This study aimed (i) to investigate the effects of free and encapsulated EO from *Rosmarinus officinalis* and *Lavandula dentata* on the biophysical parameters of excised porcine skin and (ii) to evaluate the deposition of NC-EOs on the SC of pig skin.

## 2. Results

### 2.1. Isolation and Characterization of EOs

The plants were authenticated in the Herbarium of the Faculty of Biological Sciences of the Autonomous University of Nuevo León: *Rosmarinus officinalis* (Batch 13542) and *Lavandula dentata* (Batch 030166). The EOs were extracted by the hydrodistillation technique of the aerial parts of the plants of *R. officinalis* and *L. dentata* with yield ratios (%) of 0.73 ± 0.18% (*w*/*w*) and 0.59 ± 0.22% (*w*/*w*), respectively (mean ± SD, *n* = 7). The EOs were extracted via hydrodistillation, and its constituents were identified and quantified via GC/MS and GC-FID, respectively, as described in our previous report [17].

The major components of the *R. officinalis* oil were 1,8-cineole (14.63%) and camphor (39.46%). For the *L. dentata* oil, the main components were β-pinene (11.53%) and 1,8-cineole (68.59%) [17].

To ensure quality control of the EOs, evaluating their physical characteristics, such as refractive index, relative density, and optical rotation, is important. The physical characteristics of the EOs are listed in Table 1.

### 2.2. Preparation and Characterization of the Carrier Systems

The NC-EOs were obtained using the nanoprecipitation method [18] described in Section 4.2. The physicochemical characteristics of the NC-EOs from *R. officinalis* and *L. dentata* are shown in Table 2.

The EM-EOs mean particle size and polydispersity index (PDI) for EM-*R. officinalis* were 121.11 ± 12.33 nm and 0.32 ± 0.13 PDI, respectively, while those for EM-*L. dentata* were 143.41 ± 18.50 nm and 0.40 ± 0.11 PDI, respectively. The zeta potential was −11.2 ± 0.31 mV and −23.50 ± 0.23 mV for EM-*R. officinalis* and EM-*L. dentata*, respectively. The pH of EM-*R. officinalis* was 6.28 ± 0.06, while that of EM-*L. dentata* was 6.66 ± 0.02.

### 2.3. Fourier Transform Infrared (FT-IR) Analysis

**Figure 1:**
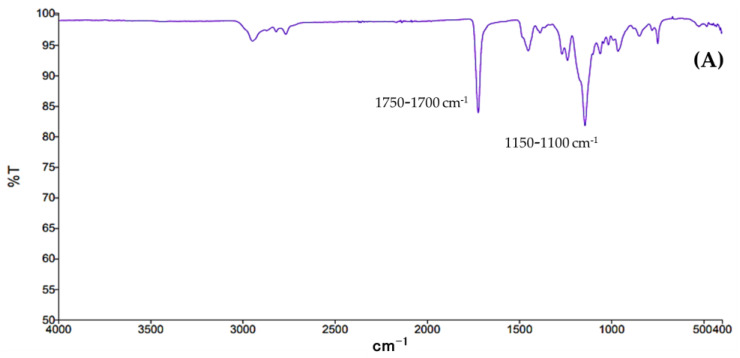

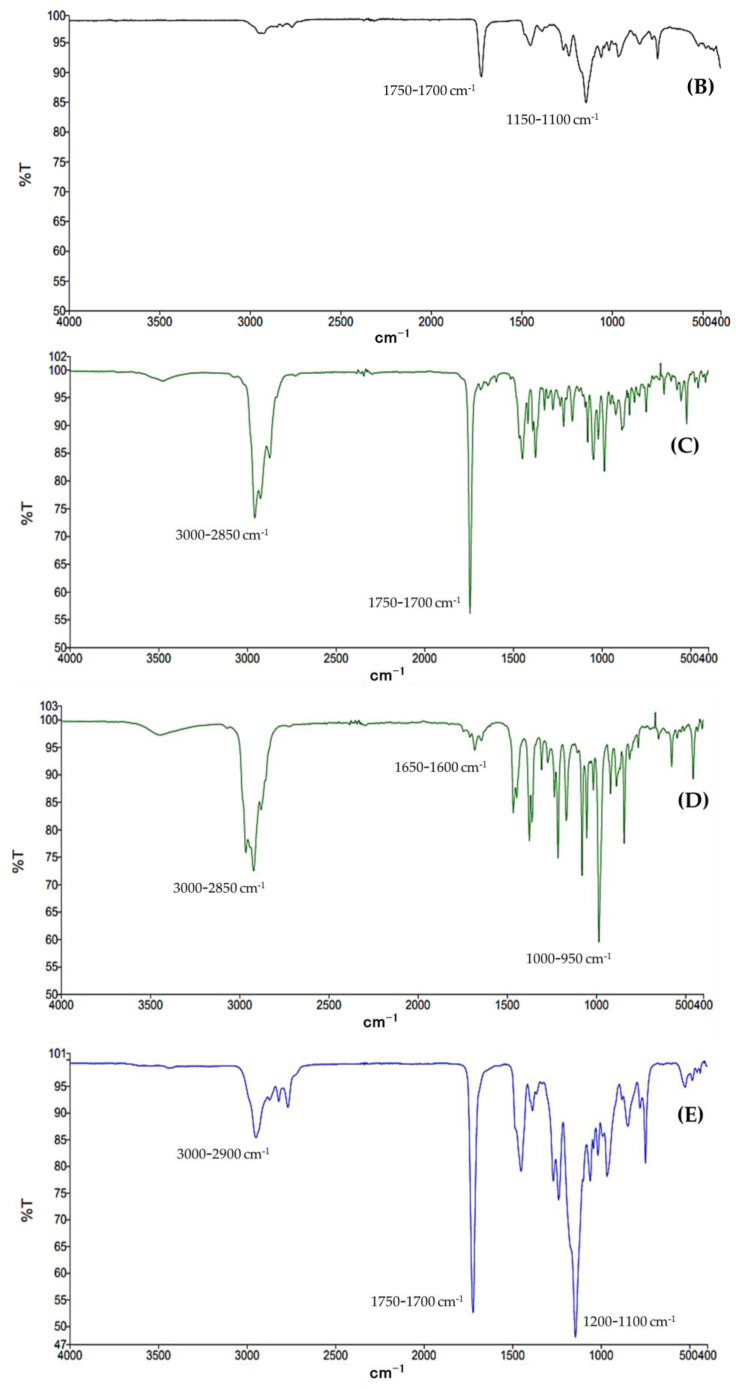

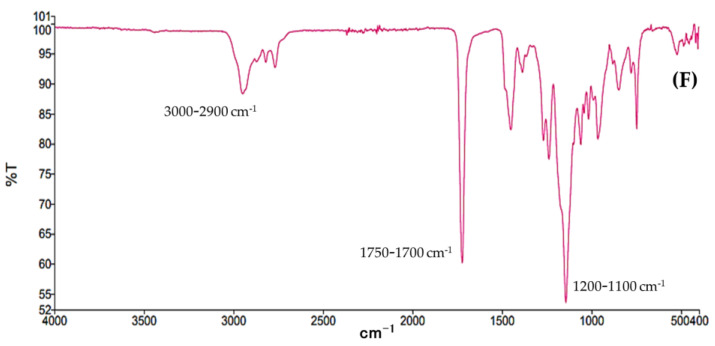
FT-IR spectra of (**A**) polymer Eudragit^®^ E PO, (**B**) NP-w/o, (**C**) EO- *R. officinalis* free, (**D**) EO-*L. dentata* free, (**E**) NC-*R. officinalis*, and (**F**) NC-*L. dentata*.

The polymer Eudragit^®^ EPO, NP-w/o, free EOs, and NC-EOs were characterized using FT-IR spectroscopy to investigate the interaction between the components of the NCs. The obtained FT-IR spectra for the polymer Eudragit^®^ EPO and NP-w/o are shown in Figure 1A and Figure 1B, respectively. The ν C=O of the carboxylic acid groups was at 1750–1700 cm^−1^ and the ν C-O of the saturated aliphatic esters was at 1150–1100 cm^−1^. The spectra of free EO-*R. officinalis* are shown in Figure 1C, with the observed ν C=O for the O-related functional groups at 1750–1700 cm^−1^. Meanwhile, in Figure 1D, for EO-*L. dentata*, it can be observed that the ν C=C of the linear alkenes was at 1650–1600 cm^−1^, and at the fingerprint region, the p C-H can be found at 1000–950 cm^−1^. In the case of both EOs, the main bands associated with the aliphatic structures were located at 2850 cm^−1^ and 2950 cm^−1^ for the aliphatic ν and ν_as_ C–H, respectively. Figure 1E,F represent the characteristic bands of the functional groups present in the polymer (1750–1700 cm^−1^ and 1150–1100 cm^−1^) of NC-*R. officinalis* and NC-*L. dentata*, respectively. 

### 2.4. Antioxidant Activity

**Figure 2:**
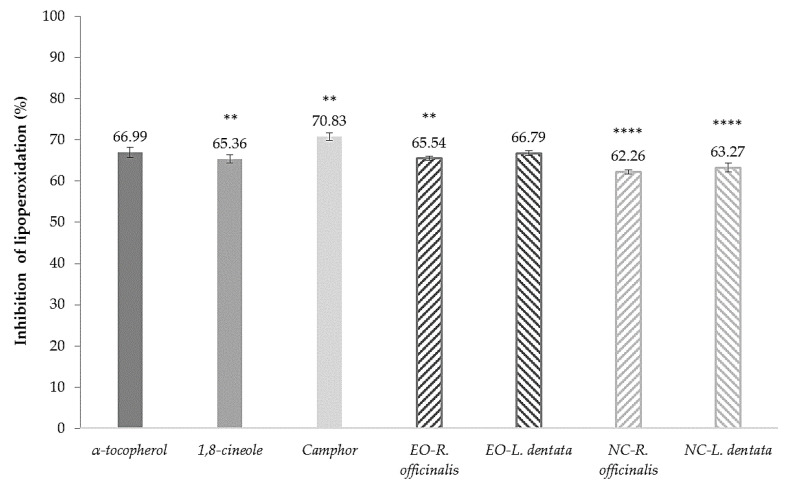
Percentage of inhibition of lipoperoxidation of controls (45 µg/mL) at 30 h of incubation time (mean ± SD, *n* = 3). * Indicates significant differences, ** *p* < 0.01, **** *p* < 0.0001, compared to α-tocopherol control.

The antioxidant activities of the control, free EOs, and NC-EOs were determined using the ferric thiocyanate (FTC) method modified in the linoleic acid system. The free and nanoencapsulated EOs showed good antioxidant activity, which was reported as the percentage inhibition of lipoperoxidation (%I). The percentages of the inhibition of lipoperoxidation at three concentrations of 15, 30, and 45 µg/mL are listed in Table 3. At the concentration of 45 µg/mL, the antioxidant activities of EO-*R. officinalis* and EO-*L. dentata* were 65.21 ± 0.60% and 66.79 ± 0.67%, respectively, closer to α-tocopherol (68.01 ± 0.59%). The significant differences in the controls, free, and nanoencapsulated EO compared with the control α-tocopherol at 45 µg/mL at 30 h of incubation time are shown in Figure 2.

### 2.5. Ex Vivo Biophysical Effect on Skin

**Figure 3:**
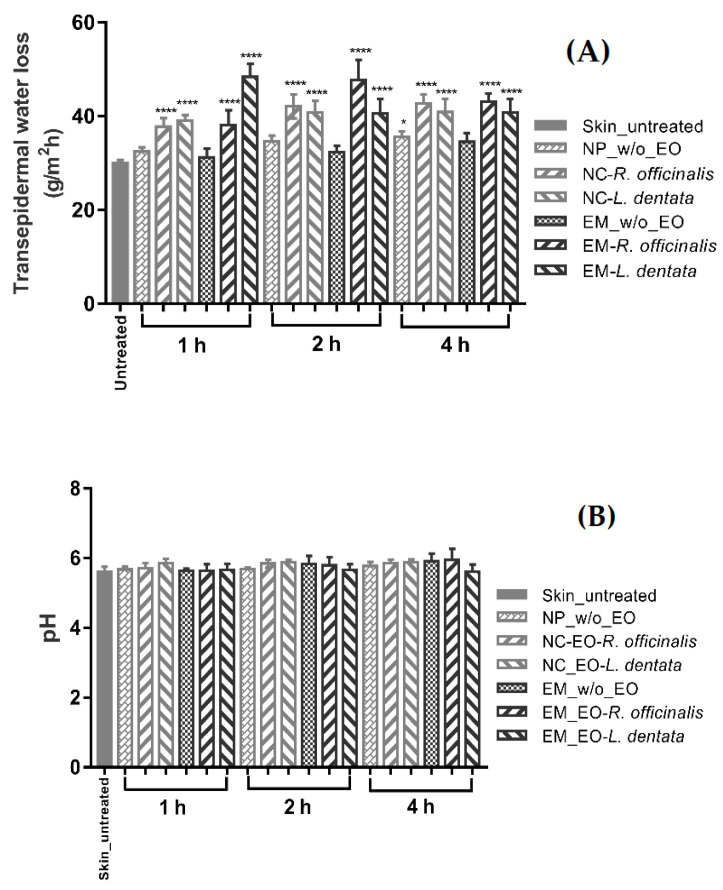

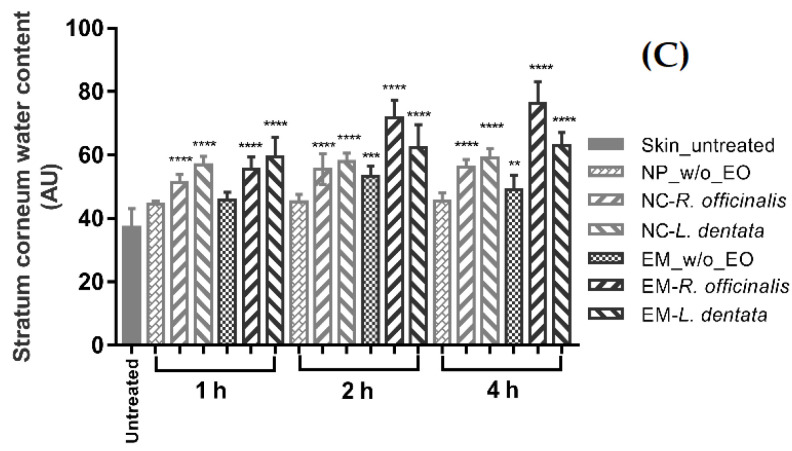
Ex vivo biophysical effect in (**A**) transepidermal water loss, (**B**) pH, and (**C**) stratum corneum water content on skin during contact time (mean ± SD, *n* = 5). * Indicates significant differences, * *p* < 0.05, ** *p* < 0.01, *** *p* < 0.001, and **** *p* < 0.0001, compared to untreated skin.

The ex vivo biophysical effects on the pig skin are shown in Figure 3. The transepidermal water loss (*TEWL*) mean value was 30.07 ± 2.20 g/m^2^ per h for the untreated skin (Figure 3A). The *TEWL* after EM-EOs skin contact was higher than that after NC-EOs contact, suggesting that free EO further altered the permeability of water in the SC. For pH, the mean values were closer than 5.7 for both the NC and EM, similar to the pH of the untreated skin (Figure 3B). The stratum corneum water content (*SCWC*) mean value for the untreated skin was 36.82 ± 2.73 AU; the *SCWC* values increased after contact with the formulations, being more significant with the NC and EM with EOs (Figure 3C).

### 2.6. Ex Vivo Deposition Studies

**Figure 4:**
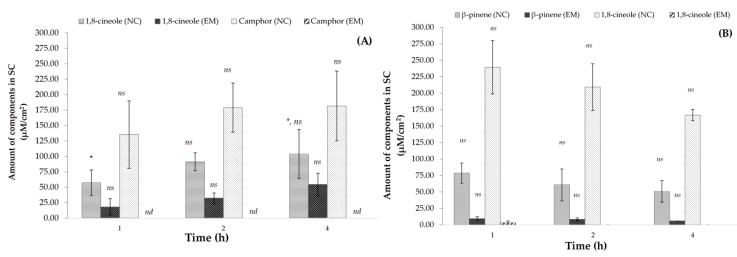
Amount of components in stratum corneum for (**A**) *R. officinalis* and (**B**) *L. dentata* at different times of skin contact (mean ± SD, *n* = 5). * Indicates significant differences *p* < 0.05 compared with 1,8-cineole in NC at 4 h. *ns*: not significant, *nd*: not detectable.

The ex vivo deposition profiles of the main components of the EO in SC are shown in Figure 4A,B for *R. officinalis* and *L. dentata*, respectively. Among the EOs, the highest deposition rate was observed for 1,8-cineole, a major component of NC-*L. dentata* after 1 h of contact time compared to NC-*R. officinalis* with a major deposition of camphor components. The EM-EO produced the lowest deposition of free EO compared to the NC-EO; for example, the rate of camphor deposition in NC-*R. officinalis* was below the quantification limit of the analytical method [17].

## 3. Discussion

In this study, free and nanoencapsulated EOs were evaluated. The physicochemical characteristics of the EOs contributed to the detection of the possible adulteration or degradation of their components, thereby ensuring their quality and biological activity for their potential dermatological application [17,19]. The physical characteristics of the EOs are listed in Table 1. The refractive index and relative density of the EO-*R. officinalis* were within the intervals established by the Pharmacopeia of the United Mexican States (FEUM) [20]. The results obtained can be compared to those obtained by Atti-Santos et al. [21] for the EOs of *R. officinalis* with an average refractive index of 1.4689, average optical rotation of +11.82°, and average specific gravity of 0.8887 g/cm^3^, which are similar to those obtained in the present investigation. With respect to EO-*L. dentata*, the results agree with those reported by El Abdali et al. [22], with a refractive index of 1.463 and a specific gravity of 0.899 g/cm^3^. The optical rotation reported for the EO was −3.0°, a value slightly higher than that obtained, which may be owing to differences in the type of soil or collection time of the *L. dentata* plant.

A comparative analysis of the extraction yield and chemical composition of the EOs was reported in a previous article [17]. The results generally showed variations due to different factors, such as species, soil conditions, geographic location, climate, and growing conditions [23]. Camphor and 1,8-cineole are components of EO-*R. officinalis,* with the highest percentage of abundance of camphor, whereas in EO-*L. dentata,* the most abundant component was 1,8-cineole, followed by β-pinene. Therefore, they were selected as the “main” components for monitoring EOs in ex vivo bioassays of pig skin.

The NC-EOs were obtained using the nanoprecipitation technique described by Fessi et al. [18]. Nanoprecipitation, also known as solvent displacement, is a highly efficient technique in biomedical research that allows for the formation of NCs with a high percentage of encapsulated hydrophobic molecules [18,24]. The NC-EO formulation was optimized by evaluating different variables, such as the type of polymer and solvent, to obtain NCs with a homogeneous particle size distribution. According to the parameters, the Eudragit^®^ E PO polymer and the organic phase composed of the mixture of acetone:isopropanol (1:1) were selected as the NC-forming polymer, as they allowed for obtaining NCs with the appropriate characteristics for its application on skin. In the NCs obtained, as the concentration of the polymer in the organic phase increased, the particle size increased. According to the foundation of this technique, when a greater number of polymer chains per unit volume of the solvent are present, the formation of NPs with a larger particle size is favored [25]. The physicochemical characteristics of NC-EOs are presented in Table 2. The average particle size of NC-*R. officinalis* and NC-*L. dentata* was >200 nm. In previous studies, NCs > 200 nm were distributed homogeneously on the SC and skin furrows, favoring their biological effects [16,26]. Owing to their size, NC-EOs can be deposited and more uniformly distributed on the SC and furrows of the skin, gradually releasing EOs in the SC [27]. Furthermore, the PDI is a parameter associated with homogeneity in the nanoparticle size distribution. PDI values close to 0 indicate homogeneous size distributions, whereas PDI values close to 1 indicate heterogeneous distributions [28]. The PDI value of both NC-EOs was close to 0.200, which indicates a homogeneous nanoparticle size and, therefore, a homogeneous distribution of NC on the SC. The zeta potential is based on the measurement of the electrostatic potential in the double electrical layer (Diffuse–Stern layer) that surrounds the NCs in dispersion. Additionally, NCs with a zeta potential greater than +30 mV or less than −30 mV generally exhibit high degrees of stability [29]. The zeta potential values for both NC-EOs were positive, with values greater than +50 mV, which is important because it could facilitate the interaction of the NC-EO with the SC, which would ensure the delivery of EOs compounds from NCs.

However, determining the EO content of NCs for the correct dosage and biological application is essential. Different investigations have reported encapsulation percentages (%E) higher than those obtained in the present study [19], which could be due to various factors, such as the type of NC-forming polymer used and the physicochemical characteristics of the encapsulated components (i.e., polarity and vapor pressure). In addition, it is important to emphasize that the encapsulation of a complex natural product, such as EO, represents greater difficulty due to its volatile nature.

Furthermore, the FT-IR spectra obtained are presented in Figure 1. The spectra of the Eudragit^®^ EPO and NP-w/o polymer (Figure 1A and Figure 1B, respectively) agree with that reported by Linares et al. [30]. A characteristic band corresponding to the carboxylic acid groups of the acrylic copolymer was observed. The principal component signals for the free EOs are shown in Figure 1C for EO-*R. officinalis*; the characteristic signals of the carbonyl groups present in the structure of the camphor component with the highest percentage abundance in the EOs were observed. Similarly, Figure 1D shows that for EO-*L. dentata*, the characteristic signal of the stretching of the functional group C=C present in the structure of β-pinene, one of the main components of EOs, was identified. For the NC-EOs shown in Figure 1E,F, the FT-IR spectra obtained for the NC-*R. officinalis* and NC-*L. dentata* were similar to those obtained for the Eudragit^®^ E PO polymer; therefore, observing the characteristic bands of the functional groups present in the polymer used was possible. This shows that there is only one chemical interaction between the NC-forming compounds, which allows us to infer the encapsulation of EOs within the NC-forming polymer [31]. In addition, bands corresponding to the formation of new compounds were not observed, indicating the stability of the NC-EOs.

Regarding the in vitro antioxidant activity, although the antioxidant capacity of EOs and their main components has been previously reported [7,32], it is necessary to evaluate whether the biological activity is preserved after nanoencapsulation; thus, by distributing the NC-EOs in the skin furrows, their potential dermatological application was retained [33].

The antioxidant activity was evaluated as the ability of EOs (free or nanoencapsulated) to inhibit in vitro lipid peroxidation in a linoleic acid model. Linoleic acid is the most abundant polyunsaturated fatty acid in the skin, and its presence ensures the health and integrity of the skin barrier, thus allowing a representative evaluation of the antioxidant activity of EOs on the skin. Briefly, the percentage of the inhibition of lipid peroxidation (%I) was determined using a modified FTC method. The FTC method measures the amount of peroxide, the primary oxidation product produced during the initial stages of lipid peroxidation. This method is based on the spectrophotometric determination of a colored complex formed by Fe^3+^ ions and ammonium thiocyanate at a wavelength of 500 nm [34]. α-tocopherol, a naturally occurring fat-soluble micronutrient with a potent antioxidant effect on the skin [35], was selected as a positive control. The major EO components, 1,8-cineole and camphor, were selected because of their abundance in EO and their previously reported potential antioxidant activity [36]. The percentage inhibition of lipoperoxidation is shown in Table 3. For α-tocopherol, the %I was directly proportional to its concentration, presenting a higher percentage of inhibition at a higher concentration. The results obtained by Gulçin et al. [37], with a %I of 54.7% for the concentration of 15 μg/mL, can be compared to those obtained by Topal et al. [38] which reported a %I of 73.88% for the concentration of 45 μg/mL, a percentage slightly higher than that obtained in this study, probably due to the modifications made to the method. Moreover, free EOs also exhibited concentration-dependent behavior. Figure 2 shows the %I at the concentration of 45 μg/mL for the EO of *L. dentata* (66.79%), which was higher than that obtained for the EOs of *R. officinalis* (65.21%). This result is comparable to that reported by Yang et al. [7] for *Lavandula* EOs, as it was more effective in inhibiting linoleic acid peroxidation in a test period of 10 days compared to other EOs, including the EOs of *R. officinalis*. Hosni et al. [39] established that the powerful antioxidant activity of EOs can be attributed to the presence of a high percentage of monoterpenes. In the present study, monoterpenes represented 62.34% and 80.71% of the EOs of *R. officinalis* and *L. dentata*, respectively, which was consistent with their demonstrated antioxidant activity (greater than 65% inhibition of lipid peroxidation). In contrast, NC-EOs showed a significantly lower %I of lipoperoxidation than free EOs with a significant difference of *p* < 0.0001 (Figure 2). This slight decrease could be related to the gradual release of the components encapsulated in the NCs into the medium. In previous reports, it was mentioned that NCs formulated via the nanoprecipitation technique behaved as biphasic release systems with a rapid initial phase, followed by a slower second phase [40]. However, the EOs maintained a %I of lipid peroxidation greater than 60%, which is noteworthy, indicating that nanoencapsulation did not significantly affect its antioxidant activity.

In contrast, the ex vivo biological tests on pig skin evaluated the effect of free and nanoencapsulated EOs on the biophysical parameters of the skin and the deposition profiles of the major EO components as a function of contact time. The pig ear skin is recognized as the most appropriate animal model because of its anatomical, histological, and physiological similarities to human skin [41]. Similarities include the epidermal thickness; hair follicle density; and glycosphingolipid, ceramide, collagen, and elastin content in the SC [42].

Biophysical parameters, such as the *TEWL*, water content, SC thickness, and pH, are the most frequently quantified parameters for assessing skin barrier function. These non-invasive evaluations are necessary to determine the effects of EOs and nanoformulations on the healthy skin barrier as an indicator of the biosafety of dermatological formulations [43]. The determination of the *TEWL*, pH, and *SCWC* using bioengineering techniques allows for the evaluation of the effects of EOs and nanoformulations on the biophysical parameters of the skin. In this sense, for the ex vivo evaluation of the effect of free EOs, emulsions (EM-EOs) were prepared. *TEWL* can be an effective marker of the function, efficiency, or integrity of the skin barrier and is commonly used as a good technique to assess structural alterations in the skin [44]. Figure 3A shows the effect of topical formulations without EOs (NP w/o-EO and EM w/o-EO) after contact with the skin surface for 1, 2, and 4 h. The *TEWL* did not show significant differences with respect to the untreated skin. In contrast, the NC-EO and EM-EO formulations showed a significant increase in the *TEWL* (*p* < 0.0001) compared to the untreated skin. This behavior has been previously reported, where the terpenes present in EOs interact with intercellular lipids, temporarily altering the reorganization of the “brick–mortar” structure of the SC, favoring the diffusion of water to the SC, and increasing the *TEWL* values, a reversible condition [45,46]. Regarding the evaluation of the pH, the acid character of the skin (pH between 4.5 and 6) is due to the so-called “acid mantle”, capable of inhibiting the growth of bacteria and maintaining the optimal acidic environment for the skin’s natural flora to thrive, thus maintaining the skin’s pH and ensuring that the acid mantle remains intact [47]. Figure 3B shows that no topical formulation presented significant differences (ns) after contact with the skin surface for 1, 2, and 4 h. This is because the formulations had pH values close to the pH range of the skin surface; thus, the contact of EOs formulations (EM-NCs) did not cause changes in the pH of the skin, that is, the acid mantle remained functional without altering the homeostasis of the skin.

In contrast, in relation to the *SCWC*, skin hydration is determined by exogenous factors, such as the temperature and relative humidity, as well as by endogenous factors, such as the natural hydration factor (FNH), formed mainly by the degradation of keratinocytes and the constituents of sweating, components capable of capturing water from the atmosphere [48]. Variations in the hydration of the SC generate changes in the dielectric constant of the skin, which is measured as a change in capacitance. Figure 3C shows the *SCWC* after contact with topical formulations with the skin surface for 1, 2, and 4 h. The *SCWC* shows significant differences (*p* < 0.0001) compared to the untreated skin; this could be because the formulations are in an aqueous medium (o/w), and when the skin is exposed to a humid environment, the FNH is capable of absorbing amounts of water that increase the *SCWC*. The physicochemical characteristics of NC-EOs, such as a size >200 nm and a positive zeta potential, favor their mechanical interaction and deposition in the SC, mainly in hair follicles and skin folds, which ensures greater interaction with the SC and increases water uptake. 

Regarding ex vivo deposition profiles, experimental studies were conducted on porcine skin in a Franz diffusion cell, and the tape-stripping technique was used to quantify the concentration of each major component of each EO in the SC [49]. The two main components of *R. officinalis* were camphor and 1,8-cineole, while those of *L. dentata* were 1,8-cineole and β-pinene, which were quantified by the previously validated GC-FID method [17]. Figure 4A shows the amount of the main components of EO in the SC (μM/cm^2^). After contact with the skin for 1, 2, and 4 h, significant differences were observed between the free EOs and nanoencapsulated EOs. The NCs favored the deposition of the main components of the EOs by up to five times that of the EMs. This could be related to the previously described physicochemical characteristics of NCs, which favor NC deposition in skin furrows and hair follicles. In addition, owing to their polymeric structure, NCs protect the components from evaporation compared to EMs. This effect was compared with that of free EOs in the emulsion, where the components presented values below the limits of quantification in the analytical methods previously developed [17].

In particular, for the NC-*R. officinalis*, the camphor component was deposited in the SC twice as much as 1,8-cineole, despite having the same %E in the NC (Figure 4A). This could be due to the physicochemical characteristics of monoterpenes, such as the partition coefficient, molecular weight, and vapor pressure. The physicochemical properties of the main components of EO-*R. officinalis*, camphor and 1,8-cineole, have a similar LogP (o/w) (2.38 and 2.74, respectively), as well as a similar molecular weight (152.23 g/mol and 154.25 g/mol, respectively). However, camphor has a lower vapor pressure than 1,8-cineole (0.87 hPa and 1.22 hPa, respectively); therefore, camphor is less volatile than 1,8-cineole and exhibits a higher permanence in the SC, which is reflected in a greater amount of camphor per cm^2^ of SC.

In contrast, for the NC*-L. dentata*, 1,8-cineole was deposited 2–3 times more than β-pinene per cm^2^ of SC (Figure 4B). This behavior can be correlated with the %E reported in the NC; 1,8-cineole has a %E of 2.89%, while β-pinene presents only a %E of 1.56% [17]. This correlates with the high number of principal components quantified in the SC. These results are important for the desired biological activity because, by depositing the main components of the EOs in greater quantities in the SC, they have a longer contact time with the skin to perform the desired biological activity.

## 4. Materials and Methods

### 4.1. Isolation and Physical Characterization of Essential Oils

The fresh plants were collected from Monterrey, N. L., Mexico, and were identified in the Herbarium of the Faculty of Biological Sciences of the Autonomous University of Nuevo León. The EOs were obtained from the aerial parts by hydrodistillation using a modified Clevenger-type apparatus. For each sample, the aerial parts of the plant were accurately weighed and then transferred to a 1000 mL round-bottomed flask with distilled water, which was connected to a Clevenger-type apparatus. The EOs were cooled to room temperature, stored in an amber bottle, and kept refrigerated until use. The EOs collected from *Rosmarinus officinalis* and *Lavandula dentata* were named EO-*R. officinalis* and EO-*L. dentata*, respectively. The EOs chemical analysis was performed by GC-MS and GC-FID [17]. The components were identified by comparing the retention indices relative to the C8-C20 n-alkanes, and the mass spectra were compared with the mass spectra from the US National Institute of Standards and Technology library.

The physical characteristics of the EOs were determined to detect possible adulteration or degradation of their components and, therefore, ensure their biological activity [17,19]. The physical characteristics determined were the refractive index, relative density, and optical rotation performed according to the Pharmacopeia of the United Mexican States (FEUM) [50]. The refractive index was determined using a refractometer (AntonPaar, Ashland, VA, USA), and the relative density was determined by using a densimeter (AntonPaar, Ashland, VA, USA). The analysis was performed three times at 25 °C. The optical rotation was determined using a polarimeter (Perkin Elmer, Waltham, MA, USA). The analysis was performed six times at 25 °C.

### 4.2. Preparation and Characterization of the Carrier Systems

**Figure 5:**
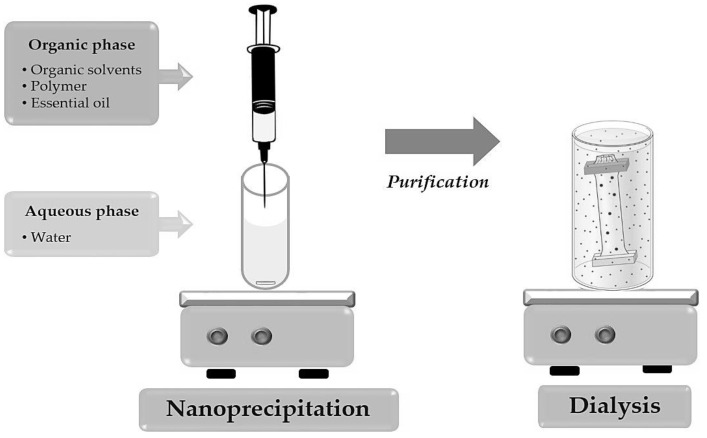
Preparation and purification of polymeric nanocapsules by nanoprecipitation and dialysis technique.

EO-loaded nanocapsules (NC-EO) were obtained by the nanoprecipitation method by Fessi et al. [18]. Briefly, an organic phase (OP), with 15 mL of solvent mixture (acetone:isopropyl alcohol (1:1)), 450 mg of Eudragit^®^ EPO, and 225 mg of EO-*R. officinalis* or EO-*L. dentata*, was incorporated into an aqueous phase (AP) of 20 mL of milliQ water under moderate magnetic stirring (125 rpm). The organic solvent mixture was removed by the dialysis technique (Figure 5). For this, the NC-EOs were placed on a regenerated cellulose membrane, under constant stirring at 350 rpm for two hours at 25 °C [51]. NPs without EOs (NP-w/o) were prepared for use as blank control samples in biological assays.

The mean size and polydispersity index (PDI) were measured by dynamic light scattering (DLS) and the zeta potential (ζ) by laser Doppler electrophoresis using a Zetasizer Nano ZS 90 (Malvern Instruments, UK), three times for each sample at 25 °C. The NCs were stored at room temperature for 8 weeks, and the stability was determined every 15 days (25 ± 2 °C) as the mean size and PDI. The encapsulation percentage (%E) of the two main components of the EO in the NC was directly determined by a previously validated GC-FID method [17] and calculated by the following formula:

(1) $$\%E=\frac{\left(mg\;of\;main\;component\;in\;encapsulated\;EO\right)}{\left(mg\;of\;polymer+mg\;of\;main\;component\;in\;total\;EO\right)}\times 100$$

An EO emulsion (EM-EOs) was prepared by adding a certain amount of EO in an aqueous solution of Tween 80 at 0.3% *w*/*v* and stirred at 20,500 rpm at 2 min with a homogenizer (VWR^®^, model VDI12, VWR West Chester, PA, USA) at room temperature. The amount of each EO in the EM was relative to the %E of the 1,8-cineol of the EO in the NCs. The EM-EOs were characterized in terms of the mean size, polydispersity index, zeta potential, and pH. EMs without EOs (EM-w/o) were also prepared for use as blank control samples in biological assays.

### 4.3. Fourier Transform Infrared Analysis

The analysis of the Eudragit^®^ EPO polymer, NP-w/o, free EOs, and NC-EOs of *R. officinalis* or *L. dentata* was performed by IR-FT spectroscopy. The polymer and free EOs were analyzed directly, while films were obtained for the NC-EOs. The analyses of the possible molecular interactions were performed with 30 scans in the 4000 to 500 cm^−1^ range with an IR-FT Optical Frontier Optical Spectrophotometer (PerkinElmer, Waltham, MA, USA).

### 4.4. Antioxidant Activity

The antioxidant activity of the free and encapsulated EOs was determined using the modified ferric thiocyanate (FTC) method [52]. The linoleic acid emulsion was prepared by mixing and homogenizing 15.5 µL of linoleic acid, 17.5 µg of Tween-20 as the emulsifier, and 10 mL of phosphate buffer (pH 7.0). For the stock solutions, 10 mg of EOs or controls (α-tocopherol, 1,8-cineole, or camphor) were dissolved in 10 mL of ethanol. Then, 5 mL of the solution containing a 15, 30, or 45 µg/mL concentration of EO or the controls solution in sodium phosphate buffer (0.04 M, pH 7.0) was added to 2.5 mL of the linoleic acid emulsion. As the negative control, a solution of 2.5 mL of the linoleic acid emulsion and 5 mL of 0.04 M potassium phosphate buffer (pH 7.0) was prepared. The solution (7.5 mL) was incubated at 37 °C, followed by the addition of 100 µL of 30% ammonium thiocyanate. Precisely 3 min after adding the 50 µL of 20 mM ferrous chloride in 3.5% hydrochloric acid, the peroxide level was determined by absorbance at 500 nm in a Genesys 10 s UV–vis spectrophotometer (ThermoLab Fisher, Plainville, MA, USA). The addition of the last solutions was repeated every 5 h until 30 h of incubation, the absorbance was determined, and the percentage inhibition values were calculated at this point (30 h). Each analysis was performed in triplicate. The percentage inhibition (%I) of the lipid peroxidation in the linoleic acid emulsion was calculated using the following equation:

(2) $$\%I=100-(\frac{A_{s}}{A_{c}}\times 100)$$

where A_c_ is the absorbance of the negative control only with the linoleic acid emulsion and sodium phosphate buffer, and A_s_ is the absorbance in the presence of EO or the standard controls [37].

### 4.5. Ex Vivo Biophysical Effect on Skin

The porcine ear was obtained from a slaughterhouse (R.E.T.S.A., Monterrey, Nuevo León, México). The full thickness of the pig skin was removed, and the tissue was stored frozen (−4 °C) for a maximum of four weeks before use. The pig skin was thawed and clamped into position between the receptor and the donor compartment of a Franz diffusion cell with an effective permeation area of 2.54 cm^2^. The receptor compartment was filled with 15 mL of phosphate-buffered saline solution (PBS) at pH 7.4 and kept under constant agitation at a temperature of 36.0 ± 0.5 °C. The donor compartment was filled with 1.0 mL of the carrier systems and covered to prevent evaporation. After the skin contact time (1, 2, and 4 h), the formulation was removed and subsequently dried with cotton. The changes in the biophysical parameters of the pig skin compared to the untreated pig skin were assessed. The transepidermal water loss (*TEWL*), skin surface pH, and SC water content (*SCWC*) were measured on the pig skin with the respective probes Tewameter TM 300, pH 905, and Corneometer CM825 attached to an MPA5 system (Courage & Khazaka, Köln, Germany). *TEWL* is a good marker of the inside–outside skin barrier and was calculated from the difference between the two measurement points using Fick’s law of diffusion and displayed in grams per hour per square meter (g/m^2^h) [53]. The pH value was directly measured. The very fine hydrophilic acidic film on the surface of the skin allows for a direct measurement by skin contact. The system measures energy changes due to the activity of hydrogen cations surrounding the very thin layer of hydrated gel at the top of the probe. The changes in voltage are displayed as pH values. The measurement of the pH level on the skin surface is an important parameter for evaluating the quality of the hydro-lipid film [53]. The SC water content (*SCWC*) of the skin surface was determined by measuring the electrical capacity of the SC. This method is based on the linear dependency of the electrical property of the epidermis to its hydration [53]. The results are displayed in arbitrary units (CM arbitrary units).

### 4.6. Ex Vivo Deposition Studies

The ex vivo deposition studies of the EO were carried out using a Franz diffusion cell. The pig ear skin was thawed and clamped into position between the donor and the receptor compartment, wherein the SC was kept in contact with the formulation and the dermis in contact with the receptor solution [26]. The donor compartment was filled with 1.0 mL of the carrier systems and covered to prevent evaporation during the 1, 2, and 4 h of skin contact time. The receptor medium consisted of phosphate-buffered saline solution (PBS) at pH 7.4, kept under constant agitation at a temperature of 36.0 ± 0.5 °C, to maintain adequate skin conditions. After the contact time, each skin sample was removed from the Franz cell and the residual formulation was removed from the skin surface prior to the tape stripping. The adhesive tape strips were prepared in advance. Further, the SC was removed by 5 successive tape strippings using Scotch tape strips (polypropylene backing and acrylic adhesive; 845 Scotch^®^ Book Tape, 3 M, Boca Raton, FL, USA). The five strips were placed individually into the tubes with 1.0 mL of methanol and then placed under stirring at 225 rpm for 30 min. The amount of the main components of EO present in the SC was determined by the GC-FID methods previously validated [17].

### 4.7. Statistical Analysis

The results are reported as the mean ± standard deviation. The statistical analysis was carried out using the single-factor one-way ANOVA test (software Graph Pad Prism version 7.0). A 0.05 level of probability (*p* < 0.05) was taken as the level of significance.

## 5. Conclusions

In this study, NC-EOs from *R. officinalis* and *L. dentata* were successfully obtained using the nanoprecipitation method. The NCs had suitable physicochemical characteristics for topical application, and the in vitro antioxidant capacity of the EOs was maintained after encapsulation. The NC-EOs favored the deposition of EOs in the SC, with independent behavior for each EO, without a permanent alteration in the structure and barrier function of the skin. These results validate the importance of the development of dermatological nanoformulations using these EOs as alternative dermoprotective agents with potential antioxidant effects to improve skin health. In summary, NCs can be used as novel prophylactic alternatives to prevent the adverse effects of oxidative stress on the skin. These advantages are the reasons for great interest in the enhanced application of NCs in the dermatological field as carriers of topical active ingredients.

## Figures and Tables

**Table 1:** Physical characterization of EOs (^1^ mean ± SD, *n* = 3; ^2^ mean ± SD, *n* = 5).

EO	Refractive Index ^1^	Relative Density (g/mL) ^1^	Optical Rotation (°) ^2^
*R. officinalis*	1.469 ± 0.000	0.894 ± 0.002	+11.80 ± 0.01
*L. dentata*	1.470 ± 0.000	0.900 ± 0.002	−1.67 ± 0.01

**Table 2:** Physicochemical characterization of nanocapsules loaded with EOs. (Mean ± SD, *n* = 3).

NC-EO	Mean Size (nm)	PDI	Zeta Potential (mV)	pH	%E	
*NC-R. officinalis*	227.73 ± 2.96	0.20 ± 0.03	54.47 ± 0.45	6.28 ± 0.06	1,8-cineole	2.95 ± 0.14
					Camphor	2.41 ± 0.13
*NC-L. dentata*	230.99 ± 8.85	0.22 ± 0.03	50.40 ± 0.75	6.66 ± 0.02	β-pinene	1.56 ± 0.13
					1,8-cineole	2.89 ± 0.12

**Table 3:** Percentages of inhibition of lipoperoxidation (%I) of free and nanoencapsulated EOs (mean ± SD, *n* = 3).

	15 µg/mL	30 µg/mL	45 µg/mL
α-tocopherol	60.83 ± 0.86	64.05 ± 0.67	68.01 ± 0.59
1,8-cineole	60.60 ± 0.81	63.99 ± 0.57	65.36 ± 1.05
Camphor	61.79 ± 0.86	64.32 ± 0.95	70.83 ± 0.90
EO-*R. officinalis*	59.85 ± 1.18	63.21 ± 1.01	65.21 ± 0.66
EO-*L. dentata*	60.21 ± 0.52	63.33 ± 0.95	66.79 ± 0.67
NC-*R. officinalis*	56.28 ± 0.72	59.97 ± 0.58	61.70 ± 0.67
NC-*L. dentata*	57.59 ± 0.82	60.57 ± 0.63	61.93 ± 0.87

## Data Availability

Not applicable.

## Abbreviation

EOs	essential oils
NC-EOs	essential oil-loaded nanocapsules
NCs	nanocapsules
ROS	reactive oxygen species
SC	stratum corneum
GC-MS	gas chromatography–mass spectrometry
GC-FID	gas chromatography with flame ionization
EM-EOs	emulsion with EOs
EM-w/o	emulsion without EOs
NP-w/o	nanoparticles without EOs
FTC	ferric thiocyanate
SCWC	stratum corneum water content
TEWL	transepidermal water loss
FNH	natural hydration factor
%E	encapsulation percentage
Log P(o/w)	octanol-water partition coefficient
PDI	polydispersity index
ζ	zeta potential
